# Phytoremediation Potential, Photosynthetic and Antioxidant Response to Arsenic-Induced Stress of *Dactylis glomerata* L. Sown on Fly Ash Deposits

**DOI:** 10.3390/plants9050657

**Published:** 2020-05-22

**Authors:** Gordana Gajić, Lola Djurdjević, Olga Kostić, Snežana Jarić, Branka Stevanović, Miroslava Mitrović, Pavle Pavlović

**Affiliations:** 1 Department of Ecology, Institute for Biological Research “Siniša Stanković”, National Institute of Republic of Serbia, University of Belgrade, Bulevar Despota Stefana 142, 11060 Belgrade, Serbia; kalac948@gmail.com (L.D.); olgak@ibiss.bg.ac.rs (O.K.); nena2000@ibiss.bg.ac.rs (S.J.); mmit@ibiss.bg.ac.rs (M.M.); ppavle@ibiss.bg.ac.rs (P.P.); 2 Department of Plant Ecology and Phytogeography, Faculty of Biology, University of Belgrade, Takovska 43, 11000 Belgrade, Serbia; vstev@eunet.rs

**Keywords:** Arsenic, fly ash, *Dactylis glomerata*, phytoremediation, photosynthesis, metabolites, oxidative stress, adaptation

## Abstract

Arsenic (As) from coal fly ash can be released into soil/groundwater, presenting a global threat to the environment and human health. To overcome this environmental problem, phytoremediation represents an urgent need, providing ‘green’ cleanup of contaminated lands. The present study focused on As concentrations in fly ash and plants, evaluation of phytoremediation potential of *Dactylis glomerata* sown on fly ash deposits together with its photosynthetic activity, and oxidative and antioxidative response to As stress. Field research was carried out on fly ash deposits at the thermal power plant “Nikola Tesla”, Obrenovac (TENT-A, Serbia) and the control site. Fly ash is characterized by alkaline pH reactions, small amounts of organic matter, a large amount of available phosphate, and total and available As concentrations. Results in this study indicate that phosphate application can ameliorate As toxicity, uptake and root-shoot transport. Furthermore, *D. glomerata* can be considered as good As phytostabilizator, because it retains more As in roots than in leaves. Excess As in leaves decreases photosynthetic efficiency (Fv/Fm) and concentrations of chlorophylls, carotenoids, and anthocyanins, whereas high content of malondialdehyde (MDA) can be a signal for biosynthesis phenolics and ascorbic acid, providing cellular redox homeostasis and recovery of photosystem II (PSII) photochemistry. In the roots, low oxidative stress under high concentrations of As is related to intense antioxidant biosynthesis. Taken together, the results in this study indicate a high adaptive potential of *D. glomerata* to As stress. These findings may suggest that physiological and metabolic tools can be used as a way forward in the ‘real field’ scenario, phytomanagement of fly ash and ecosystem services providing sustainable phytoremediation of As-contaminated sites around the globe.

## 1. Introduction

Fly ash is generated by coal combustion in thermal power plants during the production of electricity worldwide. Fly ash contains metal(loid)s, such as As, B, Cd, Cr, Cu, Mn, Mo, Ni, Pb, Se, and Zn, as well as organic pollutants which can be released in the environment [1]. Fly ash is mixed with water and pumped as slurry to surface disposal, usually formed on the surrounding land, in artificially made lagoons [2]. Dry fly ash disposed in open dumps which are not covered by vegetation presents a great treat to the environment and human health [3]. Metal(loid)s from fly ash deposits leach in the surrounding environment and can cause environmental risks for soil/groundwater entering food web [4].

Phytoremediation is defined as “green” technology that uses plants to cleanup contaminated environment from hazardous pollutants [5]. In addition, phytostabilizationis a phytoremediation technology which uses plants to reduce the mobility and bioavailability of contaminants in the environment preventing their migration in the soil/groundwater or their entry into the food chain [5]. This is achieved by using excluders which are able to immobilize pollutants in the substrate or plant roots [6]. Therefore, plants with a different combination of bioconcentration factor (BCF) and translocation factor (TF) (BCF < 1 and TF <1; BCF < 1 and TF > 1; BCF > 1 and TF < 1) show a high phytostabilization potential and represent excluders, because they possess mechanisms that maintain low uptake of soil-metal(loid) content and low shoot-metal content [7]. Furthermore, sowing a mixture of grasses and legumes and planting of shrubs and trees on fly ash deposits that are good phytostabilizators of metal(loid)s are crucial for efficient ecological restoration. These plants also provide green cover that is able to stabilize ash, prevent erosion by wind, decrease mobility and toxicity of toxic elements in the environment and provide organic matter which can bind contaminants [7,8,9,10,11,12,13,14].

Arsenic (As) in coal fly ash is present mostly as pyrite (FeAsS) and its concentration can vary from 2–1000 mg/kg depending on the concentrations in the parent coal and combustion processes [15]. Estimated mean values of As in acidic fly ash are in the range 7.4–25.0 mg/kg, whereas in alkali fly ash mean values are in the range 56.0–89.0 mg/kg [15]. According to [15,16], the mean values of As in coal fly ash in India were in the range 42.4–46.85 μg/g, whereas in Serbia the mean values of As were in the range 14.9–110.8 μg/g [9,13,17,18]. Arsenic may leach from ash into solution where it becomes mobile, reactive and toxic participating in local biogeochemical cycles [19]. Furthermore, the leaching behavior of As from coal fly ash depends on pH, redox conditions, solid-to-liquid (S/L) ratio, leaching time, Ca, Mg, sulfate, phosphate, leaching time, and temperature [15,19]. Arsenic is a metalloid that is classified as one of the most highly toxic and carcinogenic substances and according to International Agency for Research on Cancer (IARC) it is ranked as Group 1 human carcinogen. Furthermore, the Environmental Protection Agency (EPA) and Agency for Toxic Substances and Disease Registry (ATSDR) rank As at the top priority on the list of hazardous substances [20] that areassociated with skin, bladder, and lung cancer, as well as with cardiovascular diseases, neurological disorders, diabetes mellitus, and chronic kidney disease [20].

Arsenic is not an essential element for plant growth [21]. However, it can easily be taken up by plant roots in the form of arsenate (AsV) and arsenite (AIII) via transporter proteins [22,23]. Availability, acquisition, and toxicity of As in plants depends on dynamic soil reactions, its total concentration in the soil, speciation of As (AsV and AsIII), plant species, and phosphorus content in the soil [22,24]. Arsenate (AsV) is a chemical analog of phosphate and uses the same Pi channels for its entry into plant root tissue, i.e., different high-affinity Pi transporters (PHT1, phosphate transporter1) facilitate the acquisition, uptake, and translocation of Pi/AsV [25,26]. AsV tolerance is achieved by suppression of the Pi uptake system and down-regulation of PHT1 genes and WRKY transcription factors that are involved in AsV influx [27,28] as well as vacuolar phosphate transporter1 (VPT1 or AtPht1;5) that is responsible for vacuolar Pi accumulation [29,30]. Additionally, Pi fertilization can decrease uptake and accumulation of AsV in roots and enhance As tolerance in plants [31,32]. According to [33] slower rate of AsV uptake leading to the gradually activation of plant antioxidant defense system before it is overloaded by excess of As. AsIII is able to enter the root through silicon (Si) influx transporters, such as NIPs family of aquaporins (nodulin 26-like intrinsic protein OsNIP2;1/OsLs1) whereas other NIP family members, and As efflux transporters (OsLs2) are responsible for As uptake and root to shoot translocation of AsIII, respectively [34].

AsV can compete with Pi replacing it in key biochemical processes (phosphorylation of ADP to ATP in thylakoid membrane, RNA/DNA metabolism, phospholipid metabolism, glycolisis, and cellular signaling) whereas AsIII reacts with sulfhydryl group affecting catalytic functions of different proteins (structural proteins, redox regulatory enzymes, metabolic enzymes, proteolytic proteins, signal transduction proteins, and transcription factors) [22]. Arsenic in plants induces oxidative stress and produces reactive oxygen species (ROS) that together lead to lipid peroxydation and redox imbalance in the cells [22,24]. Malondialdehyde (MDA) has long been used as a lipid peroxidation marker related to oxidative stress, redox signaling [35,36] and As stress in plants [37,38]. Arsenic in plants inhibits photochemical efficiency where light harvesting complex (LHCII) and photosystem II (PSII) can be affected together with chlorophylls and carotenoids [13,24,39]. The toxic effects of As lead to a decrease of root and leaf growth, as well as wilting and violet coloration of leaves [40].

Plants require essential microelements (i.e., B, Cu, Fe, Mn, Mo, Ni, Zn) for their growth and development, and to complete their life cycles, whereas beneficial elements (Se) can act as protectors of plants against drought/metal stress stimulating root and leaf growth and increase the antioxidant potential to stress [21,41]. Essential microelements play a vital role in photosynthesis, respiration, nitrogen fixation, and antioxidant protection functioning as protein cofactors and they have a key structural/catalytic role in proteins and enzymes [21,42]. The range of optimal concentrations of elements depends on the plant species and the requirements of plants for a particular element, as well as on the concentration and availability of the elements in the environment [21,43,44,45,46]. Increases in plant element concentrations beyond the optimum (excess) reduce plant vitality and growth, i.e., toxicity occurs when an element is present in a greater amount than it needs to be for optimal functioning [21,47]. However, some elements are non-essential (e.g., As, Cd, Cr, Pb) and their presence in even low concentrations in plants can lead to toxicity and disruption of physiological processes [22] According to [44,46] plasticity in excess uptake of elements varies between plant species end elements, i.e., there are plastic variations between environments that are related to the element availability and plastic variations within plants related to the element translocations. Plants possess different mechanisms of uptake, intracellular trafficking, and accumulation in the condition of metal(loid) sufficiency/toxicity, which are responsible for the maintenance of metal(loid) ion homeostasis and tolerance in plant cells [43,48].

Plants possess several defense strategies to mitigate As stress [22,23,24]. The first step in detoxification of As in plants is reduction AsV to AsIII by the arsenate reductase (ACR), then its complexation with glutathione (GSH), phytochelations (PCs) and metallothionein (MTs) vacuole sequestration, induction antioxidant enzymes (superoxide dismutase, SOD; ascorbate peroxidase, APX; glutathione reductase, GR; catalase, CAT), and non-enzymatic antioxidants (glutathione; ascorbate; carotenoids, anthocyanins, phenolics) [22,23,24]. Ascorbate functions as an antioxidant, enzyme cofactor, participates in electron transport, reacts directly with ROS, and reduces their effect on metabolic processes [49]. In addition, the antioxidant properties of phenolics are reflected in their ability to directly remove ROS, chelating metal ions and altering the kinetics of peroxidations [50]. It is considered that flavonoids act as protectors of the PSII reaction center, removing ROS and efficiently dissipating excessive energy [51,52], whereas anthocyanins represent a class of flavonoids that possess ability to reduce potential for photooxidative damage, decrease excitation pressure and can quench ROS directly [51,53]. The framework of damage or redox signaling determine plant response to environmental stress where antioxidants function as powerful redox buffers in plant cells [54]. Therefore, the balance between ROS production and antioxidant activation, signaling, and regeneration indicate adaptive plant response to stress [54]. In this context, metabolic studies are significant to understand the chemical footprints of plants to stress response, their tolerance and signaling pathways that are essential for physiological processes [55,56].

*Dactylis glomerata* L. (cocksfoot) is a perennial tufted grass species that is widespread in Europe, Asia, and North Africa [57]. *D. glomerata* has an extensive fibrous root system. This plant species tolerates drought, heat, frost and shadow, grows on sands, loam and clay with soil pH from 5.6 to 8.4 [58]. *D. glomerata* is often planted in combination with other grasses and legumes to form ‘artificial’ meadows or reduce erosion. It is a suitable species for land reclamation, revegetation, and restoration of sites disturbed by mining [59].

We hypothesize that *D. glomerata* sown on fly ash deposits has a high phytoremediation potential for As. However, excess As in leaves would reduce photosynthetic efficiency, and the chlorophylls, carotenoids, and anthocyanins contents. Despite that, this plant is capable of activating a protective system, increasing the content of phenolics, ascorbic acid, and total radical scavenging activity in roots and leaves. The main objectives of this study were: (a) to determine As concentration in fly ash, plant roots and leaves, and relationships between As and pH, organic matter and available P_2_O_5_; (b) to evaluate the phytoremediation potential of *D. glomerata* sown on fly ash deposits; (c) to assess the kinetics of chlorophyll *a* fluorescence (ChlF) and pigments content under As excess; and (d) to evaluate the oxidative stress and antioxidative response to As stress. Findings in this study provide insight into “real field scenario”, and possible mitigation of As toxicity by phosphorus in plants. Sound knowledge related to the ecophysiological and metabolic response of plants to As stress can provide a valuable tool for sustainable ‘green’ cleanup and successful ecorestoration of As-contaminated lands.

## 2. Materials and Methods

### 2.1. Study Site

**Figure 1:**
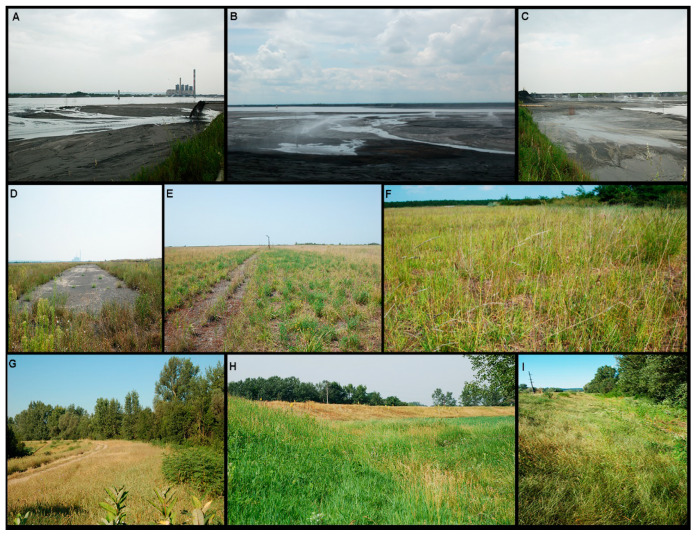
Active lagoon (thermal power plant “Nikola Tesla”, Obrenovac; TENT-A) (**A**–**C**); Lagoon 3 years old (L3) (**D**–**F**); control site (CS) (**G**–**I**).

The thermal power plant “Nikola Tesla” (TENT-A) is the biggest energy producer in Serbia and the region of South East Europe. There are 14 blocks with a total installed capacity of 3288 megawatts, which is one third of the installed capacity of “Electric Power Industry of Serbia”, and it annually produces more than 50% of Serbian electricity. TENT-A in Obrenovac (N 44°30′, E 19′58″) with six units of 1.650 megawatts of installed output power is the biggest power generation facility in the Balkans and produces 8 billion kilowatt hours per year. It is located on flat terrain on the right bank of the Sava River 40 km from Belgrade. Fly ash is collected and drained in swimming pools, and from there with hydraulic pumps it is transported to fly ash landfills. Fly ash is carried out in three lagoons (100 ha each), one of which is active (Figure 1A–C), and the other two are in the stage of temporary inactivity (passive, backup lagoons) for technical consolidation of fly ash and drainage, but also in case of accidents or termination of pouring ash.

Field studies were carried out on the passive fly ash lagoon of the thermal power plant TENT-A in Obrenovac (L3—the lagoon 3 years old, i.e., three years after the beginning of biorecultivation process (Figure 1D–F). The vegetation cover on the L3 is formed from the grass legume-mixture sown in the spring, consisting of *Vicia sativa* (60 kg/ha) and *Sorghum vulgare var. sudanese* (90 kg/ha), and perennial plants (120 kg/ha) *Festuca rubra* (35%), *Dactylis glomerata* (20%), *Poa pratensis* (10%), *Lolium multiflorum* (15%), *Medicago sativa* (10%), and *Lotus corniculatus* (10%), and in the autumn, consisting of *Vicia sativa* (50 kg/ha), *Hordeum vulgare* (100 kg/ha), and perennial plants (120 kg/ha) *Festuca rubra* (30%), *Dactylis glomerata* (20%), *Lolium multiflorum* (15%), *Lolium perenne* (15%), *Medicago sativa* (10%) and *Lotus corniculatus* (10%). The fertilization is carried out by mineral fertilizers NPK (15:15:15) which is most suitable in the amount of 1000 kg/ha. Further fertilization is carried out during the next season, in the spring by nitrogen fertilizers (KAN) in the amount of 300 kg/ha. Sown grass such as *Dactylis glomerata* is characterized by a large cover on the flat part together with *Festuca rubra* (cover with 2–100%) on the passive cassette 3 years old [14].

The control site (CS) was on the bank of the river Kolubara in Obrenovac (3 km away from the fly ash deposits of TENT-A) (Figure 1F–H). The soil at control site is sandy loam. Individuals of *D. glomerata* grow in open habitat. This site has the same climatic conditions as those on the fly ash site. The selected area is large enough to collect the same number of composite samples as on the fly ash site. Individuals of *D. glomerata* are healthy and vital with good physiological status and no visible symptoms of damage.

### 2.2. Soil, Fly Ash and Plant Analysis

The rhizosphere soil and fly ash samples were taken from the control soil and fly ash (0–20 cm). Ten composite samples of fly ash at L3 (n = 10), soil at CS (n = 10) and plant samples from fly ash (n = 10) and control site (n = 10) were collected by the quadrate method (ten quadrates of 10 × 10 m). Collected composite samples of fly ash, soil, and corresponding plants from L3 and CS were packed into plastic bags and brought to the laboratory for chemical and elemental analysis. The composite plant samples (roots and leaves) from both sites were taken for physiological, biochemical, and elemental analysis. The samples for biochemical analysis were packed into plastic bags and put in refrigerated boxes and brought to the laboratory where they were kept in a deep freezer (−80 °C) for further analysis.

The pH values (active-pH H_2_O) of the soil and fly ash samples were measured in water (fly ash: solution ratio of 1:5 *w*/*v*), with a WTW (Germany), inoLab 7110 pH meter. Organic matter was determined by calculation using the values of the total carbon (C) content in soil and fly ash that was previously determined by the [60] Available phosphorus (P_2_O_5_, mg/100 g) was extracted with ammonium acetate-lactate (A-L solution, pH 3.7, ratio 1:20) and determined by flame photometry [61].

The total As, B, Cu, Mo and Se concentrations in fly ash and soil as well as in roots and leaves were determined according to methods 3051 [62] and 3052 [63], respectively. The roots and leaves of plants were separated, washed with tap and deionized water, oven dried at 80 °C for 12 h, and homogenized by grinding in a stainless steel blender and then passed through sieves of 2 mm mesh size. Preparation of all samples was done by wet digestion in a microwave oven (CEM, MDS-39 2000). Plant available fractions of As, Cu, Mo, and Se in fly ash and soil were determined by using diethylene-triamine-pentaacetic acid (DTPA) according to the method found in [64]. Available B content in fly ash and soil was determined in hot water. Analytical procedures were tested using certified reference materials: fly ash (ash from coal BCR—038), soil (clay, ERM—CC141), and grass (*Lolium perenne* L., ERM—CD281) were provided by the Institute for Reference Materials and Measurements (IRM) and approved and certified by the European Commission Joint Research Center (JCR), in Belgium. As, B, Cu, Mo, and Se concentrations in fly ash, soil and plant material were determined using ICP-OES (Inductively coupled plasma—optical emission spectrometry, Spectro Genesis, Kleve, Germany), and expressed in mg/g of dry weight. According to [65] the bioconcentration factor (BCF) and translocation factor (TF) were calculated as follows [1,2], respectively:

BCF = [element] in root/element] in soils, fly ash(1)

as a ratio of element concentration in the root and element concentration in the soil/fly ash.

TF = [element] in leaf/[element] in root(2)

as a ratio of element concentration in the leaf and element concentration in the root.

### 2.3. Chlorophyll a Fluorescence

The kinetics of chlorophyll *a* fluorescence (ChlF) of leaves were measured under field conditions using a portable fluorimeter (Plant Stress Meter, BioMonitor SCI AB, Sweeden) according to [66]. The leaves were adapted to dark for 30 min after which the chlorophyll excited 2s actinic light density of 200–400 µmol photons m^−2^s^−1^. The parameters of chlorophyll *a* fluorescence were measured: Fo (the minimum of fluorescence in the dark, when all PSII reaction centers and electron acceptors are fully oxidized, hence “open” for photochemistry); Fm (the maximum of fluorescence in the dark, when all PSII electron acceptors are fully reduced, preventing photochemistry, i.e., all active PSII are “closed”); Fv (variable fluorescence); t_1/2_ (half the time required to reach maximum fluorescence from Fo to Fm); Fv/Fm (the maximum quantum efficiency of PSII photochemistry) and the ratio was correlated with the number of functionally active PSII reaction centers).

### 2.4. Metabolite Analysis

#### 2.4.1. Pigment Content

Chlorophylls and total carotenoids were extracted with 80% acetone and the absorbance of the samples was measured at 663 nm, 645 nm and 480 nm using a spectrophotometer (UV–vis spectrophotometer, Shimadzu UV-160). Chlorophylls (Chl *a*, Chl *b*, and Chl *a+b*) and total carotenoids (Tot Carot) content was calculated using equations according to [67,68], respectively and expressed as mg/g of dry weight. The anthocyanins in leaves were determined according to [69,70]. The pieces of leaves were placed in 1 mL DMSO and heated for 2 h at 65 °C, then 0.5 mL of 2 N HCl was added, and heated for 4 h at 65 °C. The absorbance of the samples was measured at 650 nm, 620 nm, and 520 nm spectrophotometrically. The amount of anthocyanins was expressed as mg/g of dry weight.

#### 2.4.2. MDA Content

The content of malondialdehyde (MDA) in the leaves and roots was measured according to [71]. Samples (0.5 g) were homogenized in 5 mL of 80% ethanol, containing 0.05 mL of 2% butylated hydroxytoluene. A solution of 1 mL of the supernatant, 0.5 mL of 0.65% thiobarbituric acid and 0.5 mL of 10% trihloracetic acid was heated for 15 min at 95 °C. Then cooled on ice and centrifuged for 10 min at 3000× *g*. The absorbance of the extract was measured spectrophotometrically at 450 nm, 532 nm, and 600 nm. The amount of MDA was expressed as nmol g^−1^ of fresh weight.

#### 2.4.3. Phenolic Content

Phenolics (Free Ph—free phenolics, highly soluble fractions) were extracted from leaves and roots with 80% (*v*/*v*) boiling aqueous methanol solution followed by ethyl acetate. Bound phenolics (Bound Ph—fractions of phenols which are either esters, or bound to the polysaccharide matrices of the cell wall, or polymerized into lignin) were prepared by boiling the insoluble residue from the above procedure in 2N HCl for 60 min and transferred to ethyl acetate. Total phenolics (Tot Ph, free and bound phenolics) were determined according to [72] The absorbance of free and bound phenolics was measured at 660 nm spectrophotometrically according to [73]. A standard curve was constructed with different concentrations of ferulic acid (Serva, Heidelberg, Germany). Concentrations of phenolics were expressed as mg/g of dry weight.

#### 2.4.4. Ascorbic Acid Content

Concentrations of ascorbic acid (AsA) in plant leaves and roots was determined according to the method in [74]. Samples (0.5 g) were homogenized in 5 mL of 5% metaphosphoric acid, containing 5 mL of ethyl acetate. A solution of 1 mL of extract, 1 mL ferroamonium sulfate, 0.2 mL HCl, 1 mL quinaldic acid, and 1 mL of pyridine was heated until the appearance of pink color. Then, AsA was extracted with 9 mL of chloroform (30 s). The absorbance of the solution was measured spectrophotometrically at 380 nm and concentrations of AsA expressed as mg/g of fresh weight.

#### 2.4.5. Radical Scavenging Activity

The antioxidant capacity in plant leaves and roots was determined by using free radical 1,1-diphenyl-2-picrylhydrazyl (DPPH) according to [75]. Samples (0.5 g) were homogenized in 10 mL of 95% ethanol. Each sample was done in three increasing concentrations (5, 25 and 50 µL) with the addition of 0.5 mL of DPPH. The absorbance was measured at 517 nm spectrophotometrically. Low absorbance of the reaction solution indicates high antioxidant activity, as can be seen from the color changes of the solution from purple to yellow. Radical scavenging activity of each extract was calculated using the formula [3]:

RSCA (%) = [(Ao − A1)/Ao] × 100(3)

where Ao is the absorbance of the control and A1 is the absorbance of the antioxidant in the sample solution.

### 2.5. Statistical Analysis

Values are presented as a mean (M) of ten replicates for chemical analysis of fly ash and soil and As concentrations in plant leaves and roots as well as for the physiological and metabolite analysis of plant material with standard deviation (SD). The differences between groups were determined by analysis of variance (ANOVA) and Student *t* test. The relationship between parameters was determined by Pearson correlation coefficients (r). Statistical analysis was performed by using the package Statistica 7.0 (StatSoft In., Tulsa, OK, USA, 2004).

## 3. Results

### 3.1. Chemical Characterization of Soil/Fly Ash and Element Concentration

Chemical properties, total and available element concentrations in soil and fly ash, element concentrations in plant roots and leaves, bioconcentration factor (BCF), and translocation factor (TF) of *D. glomerata* growing on the soil at the control site (CS) and the fly ash site (L3) are presented in Table 1. Results show higher values of pH in fly ash than in soil. Furthermore, fly ash showed alkaline reaction whereas the soil was neutral. Organic matter content was significantly higher in soil at CS than in fly ash at the L3 site. However, available content of P_2_O_5_ was higher in fly ash than in soil due to fertilization. The total and available As, B, Cu, Mo, and Se concentrations in fly ash were significantly higher than in soil. The total As, B, Cu, Mo, and Se concentrations in fly ash were toxic [40]. However, total As, Cu, Mo and Se concentrations in the soil at the control site were within the range of average values for soils, while the total B concentrations were deficient [40].

Results in this study show higher values of As, B, Cu, Mo and Se concentrations in roots and leaves of *D. glomerata* sown on fly ash deposits versus soil, except Cu concentrations in leaves at the fly ash site. Leaf As concentrations of *D. glomerata* from fly ash site were toxic whereas B, Cu, Mo and Se concentrations in leaves were within optimal range for plants [40]. In addition, leaf As, Cu, Mo, and Se from control site were within optimal range for plants, except deficient B concentrations [40]. The BCF for As (L3, CS), B (L3), Cu (L3, CS) and Mo (L3, CS) were lower than one (BCF < 1), whereas for B (CS) and Se (L3, CS) were higher than one (BCF > 1). The TF for As (L3, CS), B (CS), Cu (L3) and Se (L3, CS) were lower than one (TF < 1) while for B (L3), Cu (CS) and Mo (L3, CS) were higher than one (TF >1). In addition, the BCF for As, B, Mo, and Se were significantly higher at the fly ash site compared to the control site, except for Cu. However, the TF for As and Cu were significantly lower, and B significantly higher, at the fly ash site compared to the control site, whereas TF values for Mo and Se between the fly ash site and control site were not significant.

### 3.2. Chlorophyll a Fluorescence and Pigments

Clorophyll *a* fluorescence parameters and pigment content in the leaves of *D. glomerata* at the control site (CS) and the fly ash site (L3) are presented in Table 2. Values of Fm, Fv, Fv/Fm, and Fm/Fo were significantly lower at the fly ash site in comparison to the control site. The values of Fv/Fm and the ratio of Fm/Fo in *D. glomerata* sown on fly ash deposits were below optimal range for plants (0.750–0.850; 5.0–6.0) [76] indicating low vitality of plants growing on fly ash. However, there was no significant difference in values of Fo between fly ash and control site, whereas the t_1/2_ values from the fly ash site were significantly higher than at the control site. The content of Chl *a*, Chl *b*, Chl *a+b*, the ratio of Chl *a/b*, content of total carotenoids and anthocyanins were significantly lower in *D. glomerata* at the fly ash site than at the control site.

### 3.3. Oxidative Stress and Antioxidant Protection

Concentrations of malondialdehyde (MDA), phenolics (Free phenolics, Bound phenolics, Total phenolics), ascorbic acids (AsA), and radical scavenging activity (DPPH) in leaves and roots of *D. glomerata* at the control site (CS) and the fly ash site (L3) are presented in Table 2. MDA content in the leaves of *D. glomerata* sown on fly ash deposits was significantly higher compared to the control site, whereas in the roots there were no significant differences in MDA content between sites. The content of free, bound, and total phenolics as well as the content of ascorbic acids were significantly higher in the leaves and the roots of *D. glomerata* sown on fly ash deposits compared to the control site indicating activation of antioxidant mechanisms in a stress condition. However, DPPH radical scavenging activity was higher in the roots, and lower in the leaves of *D. glomerata* sown on fly ash deposits, compared to the control site.

### 3.4. Relationship between Chemical Properties, As Concentrations, Photosynthetic Parameters, Oxidative Stressand Antioxidative Capacity

The relationship between parameters of chemical properties of fly ash and soil, total and available As concentrations in fly ash and soil, and As concentrations in roots and leaves of *D. glomerata* on fly ash deposits and soil on the control site is presented in Table 3. Results in this study show positive correlations between pH and organic matter, total and available As concentrations, between organic matter and total and available As concentrations, and between total and available As concentrations in fly ash whereas negative correlations were noted between P_2_O_5_ and pH, organic matter, total and available As concentrations in fly ash. Furthermore, positive correlations were noted between pH, organic matter and As concentrations in root and leaves, between total and available As concentrations in fly ash, and between As concentrations in root and leaves. However, negative correlations between P_2_O_5_ and As concentrations in leaves and roots as well as between P_2_O_5_ and BCF, TF may indicate an ameliorative effect of P_2_O_5_ on As uptake and translocation in plants. Positive correlation was noted between the As concentration in roots and the As concentration in leaves. However, at the control site, significant negative correlations were found only between available As concentrations in soil and pH, and organic matter.

Negative correlations were found between As concentrations in the leaves of *D. glomerata* sown on fly ash deposits and values of Fv/Fm, Fm, Fv, Chl *a*, Chl *b*, Chl *a+b*, total carotenoids and anthocyanins (Table 4) indicating that the photosynthetic efficiency and concentration of pigments were affected by excess As concentrations in leaves. However, positive correlations were found between As concentrations in leaves and values of Fo, Fm/Fo, t_1/2_ and Chl *a/b* (Table 4). Furthermore, positive correlations were noted between As concentrations in leaves and the content of MDA, phenolics, AsA, and DPPH antioxidant activity (Table 4). Negative correlation was found between As concentrations in the root of *D. glomerata* sown on fly ash deposits and MDA content, whereas positive correlations were found between As concentrations in the root and the content of phenolics, AsA, and DPPH antioxidant activity (Table 4). However, at the control site correlations between As concentrations in leaves and chlorophyll *a* fluorescence parameters, content of pigments, MDA, and antioxidants, as well as between As concentrations in roots and content of MDA and antioxidants, were not significant (Table 4), indicating that As content in plants did not induce oxidative stress that can decrease photosynthetic efficiency and content of pigments, whereas antioxidants remained efficient in order to maintain metabolic homeostasis in plant cells.

The relationship between chlorophyll *a* fluorescence parameters, content of pigments, MDA, and antioxidants in the leaves of *D. glomerata* sown on fly ash deposits is presented in Tables S1 and S2. Positive correlations between pigments and the values of Fm, Fv, and Fv/Fm, together with negative correlations between pigments and MDA show that As-induced oxidative stress may decrease pigments content and photosynthetic efficiency. However, positive correlations between some chlorophyll *a* fluorescence parameters and antioxidants, and between antioxidants themselves suggest activation of protection mechanisms in the leaves. In the roots of *D. glomerata* sown on fly ash deposits, negative correlations were noted between antioxidants and MDA content, whereas positive correlations were found between antioxidants (Table S3).

## 4. Discussion

### 4.1. Chemical Properties of Soil/Fly Ash

In this study, the pH of the fly ash was alkaline, which is in accordance with the results of [77] (7.7–7.9), [10] (8.85), [13] (7.95) and [18] (7.78). The organic matter content was lower in fly ash than in soil (1.9-fold) because it takes much longer than 3 years for the plants to provide a high amount of organic matter. In this study, higher content of available P_2_O_5_ in fly ash than in soil (152%) is associated with application of chemical P fertilizers. Similar values of available P_2_O_5_ in fly ash have been reported by [13] (19.2 mg/100 g) and [18] (25.3 mg/100 g). Generally, phosphorus is characterized by low mobility in soil and low availability for plant uptake due to high fixation for soil matrix, such as clay minerals, Al/Fe oxides and Ca/Al/Fe phosphates from which P can be released by dissolution and desorption processes [78]. Negative correlation between organic matter and available P_2_O_5_ (r = −0.90) may suggest more plant-available P due to low content of organic matter in fly ash which provides a low number of adsorption sites. Furthermore, a major form of P fertilizers includes a monocalcium phosphate (MCP) that after application to soil undergoes a wetting process that generates dicalcium phosphate (DCP) which is available to plants [78]. Negative correlation between P_2_O_5_ and pH (r = −0.82) indicates that with decreasing of pH rhizosphere solution, the available P increases and according to [78] rhizosphere acidification can lead to efficient mobilization of P from soil and its acquisition and uptake by plants.

### 4.2. Element Concentrations in Soil/Fly Ash/Plant

Results in the present study show higher total and available B concentrations in fly ash compared to soil from the control site (11-fold and 4-fold higher, respectively). However, the total B concentrations in fly ash (L3) were lower than those found in fly ash generated by the combustion of lignite (320–1900 µg/g) [79]. According to [40], low values of B can be found in sandy and clayey soils, as well as in podzol and fluvisol, which agrees with our results at the control site. Furthermore, the available B concentrations in this study were less than the amount of water-soluble B in fly ash obtained from thermal power plants in Australia (2.5–13.7 µg/g) [80] and Japan (4.0–14.0 µg/g) [81]. Despite the higher available B concentrations in fly ash compared to soil, the fraction of available B concentration relative to the total B content in fly ash, is smaller (3%) than that in soil at the control site (7%) due to strong binding of B to the hydroxides of Al and Fe, alumosilicates and CaCO_3_ in fly ash [40] and as a result of that the amount of B that *D. glomerata* can be taken up is sufficient to maintain normal B content in the leaves. Results in this study show that at the fly ash site, *D. glomerata* has BCF < 1 and TF > 1 for B indicating greater amounts of B in the leaves than in the roots. High concentrations of B in leaves of *D. glomerata* at the fly ash site could be related to the BOR2 and BOR4 transporters in roots, which export B to xylem and further to the leaves under condition of high B concentrations in soil [82]. B export from root cells to xylem reduces the concentration of B in the roots by up to 30–75% [83]. In leaves, the high concentration of B can be redistributed from simplast to apoplast, where B can bind to the cell wall [84]. It has also been found that B is readily soluble in water, it can be quickly washed off by rain from the leaves, which reduces B concentrations to the level that is optimal for the plants functioning [84].

Total Cu concentrations were higher (316%) while available Cu concentrations in fly ash were lower (70%) than those in soil. Similar total Cu concentrations at L3 fly ash site (TENT-A in Obrenovac) were measured (19.8–58.0 µg/g) [17]. In this study, available Cu concentrations were lower in fly ash compared to soil (70%) and the fraction of available Cu concentration relative to the total Cu content in fly ash is smaller (2%) than that in soil at the control site (8%). According to [85] in alkaline fly ash extractable Cu can vary in the range 0.5–3% which is in line with our results. Reduced mobility and availability of Cu to plants is associated with organic matter (r = −0.89), alkaline reaction (r = −0.80) and high carbonate content in fly ash [40,86]. In the present study, the content of organic matter is generally low in fly ash, and according to [87] low organic matter content is quite enough to reduce Cu mobility by up to 77%. Results in this study show that at the fly ash site higher amounts of Cu are retained in the roots than in the leaves compared to the control site (161% higher and 73% lower, respectively). Similar results were obtained for *Pistacia terebinthus* and *Cistus creticus* grown on Cu mine tailings [88]. The higher amount of Cu in the roots could be related to stronger binding capacity of Cu to the root cell wall (60–93%) which reduces its further transport to the leaves [89]. At the fly ash site it was noted that the BCF < 1 and TF < 1 for Cu. The same results were obtained in other plants grown on fly ash such as *Ricinus communis, Ipomea carnea* [90], *Saccharum munja* [91] and *Festuca rubra* [13]. The Cu homeostasis in the plant cell is regulated by mechanisms which are able to maintain the amount of Cu that is optimal for cellular processes [92]. Despite limited Cu translocation capacity from roots to leaves, *D. glomerata* at the fly ash site has Cu concentrations in leaves that are in the optimal range, which could suggest efficient regulation, synthesis, and activation of various transporters and shaperones in sequestration and transportation of Cu that maintain Cu homeostasis in plant cells.

Total and available Mo concentrations in fly ash were higher compared to the control site (162% and 300%, respectively). Total Mo concentrations in fly ash were less than those measured for fly ash (7–117 µg/g) [93]. Similar to our results, total Mo concentrations in fly ash (L3, TENT-A in Obrenovac) ranged from 0.3–2.7 µg/g [17]. Furthermore, available Mo concentrations in fly ash can be 1.7 mg/kg [94] which is consistent with our results. Mo shows the highest solubility in the alkaline medium of fly ash [40,86,95,96] and our results confirmed it (r = 0.93). However, despite the higher fraction of available Mo concentration relative to the total Mo content in fly ash (1.3%) than that in soil (0.7%), available Mo content was still small, which could be related with its sorption on Fe and Al hydroxides (86). In this study, Mo content in roots and leaves of *D. glomerata* sown on fly ash is higher compared to the control site (1.2% and 118%, respectively). According to [97] large amounts of Mo in plants grow on slightly alkaline soils, while those concentrations in the same plants on acidic soil or soil with lower amounts of Mo were lower which is in accordance with our results. In addition, on both sites, BCF <1 and TF >1 for Mo indicating a larger amount of Mo in the leaves than in the roots which can be related to MOT1 and MOT2 transporters that have been found to play an essential role in its absorption [98], intracellular transport, allocation between roots and leaves functioning as regulators of total Mo accumulation in the plant and in the maintenance of its homeostasis in plant cells [99]. Due to generally low available Mo concentrations in fly ash, the amount of Mo that *D. glomerata* can absorb by roots (r = 0.44) and transport to the leaves (r = 0.89) did not exceed the optimal range in leaves.

Results in the present study show higher total (9-fold) and available Se concentrations (1.7-fold) in fly ash compared to soil. Higher total Se concentrations than ours were measured in the fly ash obtained from the Miliken Staton thermal power plant, NY, USA (5.1 µg/g) [100] and in the fly ash from the thermal power plant in the Netherlands (16.1 µg/g) [101]. Furthermore, average Se concentrations in soils is 0.4 mg/kg [102]. According to [40] the concentrations of Se in clay soils and loam can be high which is in line with our results. According to [86] Se mobility and solubility increase with higher pH which our results confirmed (r = 0.93). Despite the higher available Se concentrations in fly ash, the fraction of available Se concentration relative to the total Se content in fly ash was lower (1.4%) than that in soil (8%), which could be associated with strong Se affinity for Al and Fe hydroxides [86]. In this study, Se concentrations in roots (217%) and leaves (214%) of *D. glomerata* sown at fly ash deposits were higher compared to the control site. Most grasses and crops when grown on soil enriched with Se contain <25 µg/g Se in their tissues [103]. For Se at both sites, BCF > 1 and TF < 1 were noted, indicating a significant accumulation of Se in the roots, whereas a smaller amount of Se is transported to the leaves. Selenium uptake and accumulation in plants depends on the plant species, Se concentrations, and Se speciation (selenate, selenite, selenide, SeCys, SeMet) [41,104,105]. Selenate is the dominant form in alkaline soils, while selenite and selenide are the dominant forms of Se in neutral or acidic environments [40,41]. Selenate is transported inside the plant root through sulfate transporters (SULTR1;2) and then it is translocated through xylem to the leaves, where it is metabolized in plastids via sulfur enzymes to SeCys and SeMet which can be non-specifically incorporated into proteins and can cause toxicity in plants [106,107,108] or can be further converted in methylated form of SeCys and SeMet that can confer Se tolerance, i.e., they can volatize into atmosphere in non-toxic form dimethyldiselenide (DMDSe) [108]. According to [109], non-accumulator plants that are enriched with Se can sequester it and accumulate to safe levels in vacuole of mesophyll cells of leaves.

### 4.3. As Concentrations in Soil/Fly Ash/Plant

Results in this study show higher total As concentrations in fly ash versus soil (276%). Total As concentrations in fly ash were toxic and they were in the range obtained by other authors, such as [9] (110.8 μg/g), [13] (18.32 μg/g), [17] (14.9–83.2 μg/g) and [18] (21.62 μg/g). Different As concentrations in fly ash could be due to differences in coal quality, combustion conditions, differences in operating parameters of blocks in thermal power plants (flue gas purification equipment) and transporting systems through which fly ash is being transported from the thermal power plant to the fly ash deposits [14]. Higher available As concentrations in fly ash than in soil (226%) indicate increased mobility and solubility of As in fly ash, which can be related to high total As concentrations (r = 0.95), and alkaline reaction of fly ash (r = 0.95), as well as increased amounts of soluble organic matter (r = 0.96) [110,111]. According to [110] in the alkaline conditions about 80% of humates in fly ash can be quickly and easily released into the solution. Dissolved organic anions can inhibit As sorption on the surface of ferrohydrates and form strong complexes with them, increasing the amount of dissolved As ions in the soil solution [112]. Furthermore, As and P are chemical analogues and due to similar chemical behavior they can be substituted for the same chemical reactions and adsorption sites [113]. Our results show negative correlations between P_2_O_5_ and total and available As concentrations in fly ash (r = −0.84 and r = −0.76, respectively) suggesting an increase of As concentrations in fly ash with phosphate amendment. Increased available As concentrations after P amendment in soils have been reported by other authors who indicated that with high concentrations of P amendment, all P in the soil solution was adsorbed onto soil surface, i.e., all adsorption sites became saturated by P leading to greater As availability [113,114]. At the control site, with increased pH values and organic matter, the available As concentrations decrease leading to the small As content in the roots and leaves of *D. glomerata*. Biogeochemical properties of As in plant-soil system related to pH is different from fly ash, i.e., under high soil pH, As has lower mobility and availability compared to fly ash [40,86,95]. Furthermore, As is quickly adsorbed to soil organic matter which leads to smaller As available content [40].

Higher concentrations of As in the roots and leaves of *D. glomerata* sown on fly ash deposits versus the control site (182% and 203%, respectively) can be associated with high total and available As concentrations in fly ash (r = 0.93 and r = 0.94; r = 0.80 and r = 0.78, respectively). Furthermore, As concentrations in the leaves of *D. glomerata* sown on fly ash deposits were toxic [40]. Plants growing on soil contaminated with As contain high As concentrations in their tissues [115], which agrees with our results. Similar results have been reported from [13] who found high As concentrations in the roots (7.59 μg/g) and leaves (6.08 μg/g) of *Festuca rubra* sown on fly ash deposits (L3, TENT-A in Obrenovac). However, lower concentrations of As than ours were found in the leaves of *Calamagrostis epigejos* (2.71 µg/g) and *F. rubra* (2.66 µg/g) growing on the fly ash deposits of TENT-A in Obrenovac [9]. Generally, higher As concentrations were found in the plant roots than in the leaves [116], which was consistent with our results. Higher As concentrations in the roots than in the leaves of *D. glomerata* sown on fly ash deposits may be the result of enhanced synthesis the arsenate reductase (ACR) enzyme, which reduces As (V) to As (III) by using GSH as reductant. Thus, [116] have shown that in 10 species of herbaceous plants As is present as As (III), and that the plants are protected against oxidative phosphorylation by reducing and accumulating As as arsenite (As III). Except for the ACR enzyme, other enzymes also show As(V) reductase activity, such as glutaredoxins (GRXs), glyceraldehyde-3-phosphate dehydrogenase (GAPDH), polynucleotide phosphorylase, purine nucleoside phosphorylase (PNP) [22]. As(III) inside the plant roots form complexes with GSH, PCs and MTs, and they are then sequestered in vacuoles [23,117]. More As accumulated in the roots can be due to over-expression of transcription factor *OsARM1* (ARSENITE-RESPONSIVE MYB1), which leads to the down-regulation of expression of *OsLsi2* and *NRAMP1* transporters that are responsible for xylem loading and transport from root to shoot [118]. Over-expression of transporters *OsNIP1;1* and *OsNIP3;3* localized on the plasma membrane can restrict As loading into xylem and decreased As accumulation in shoots (41–56%) [119]. Furthermore, disruption of inositol transporters (AtINT2 and AtINT4) can decrease As concentrations in phloem and subsequently decrease As content in leaves [120]. Also, As(III) antiporter *PvACR3;1* mediated in it sequestration into vacuoles increasing As concentrations in roots (14–29%) and decreasing its content in shoots (55–61%) [121]. Knockout of *OsABCC1* transporter (ATP-binding cassette, ABC) can limit As transport and accumulation in the shoots [122].

In *D. glomerata* sown on fly ash deposit, BCF <1 and TF <1, indicating that a larger amount of As is retained in the roots and smaller amount is transported to the leaves pointing to the fact that *D. glomerata* is an excluder plant with a high potential for phytostabilization and phytoremediation of As in fly ash. Similar results were found in plants growing on fly ash, such as *Thelypteris dentate* [123], *Jatropha curcas* [124], and *Festuca rubra* [13]. Results in this study show lower values of BCF and TF for *D. glomerata* sown on fly ash deposits compared to the control site (48% and 79%, respectively). Furthermore, negative correlations between P_2_O_5_ and As concentrations in roots and leaves (r = −0.92 and r = −0.93, respectively) and between P_2_O_5_ and BCF and TF (r = −0.74 and r = −0.85, respectively) may indicate that phosphate application may reduce the As uptake by roots and its translocation to the leaves. Amelioration of arsenic toxicity by phosphate application was reported by other authors who found that high phosphate treatment reduced As toxicity in *Vigna radiata* [125] and *Vigna mungo* [126], decreased uptake of As by roots, and suppressed As root–shoot transfer [24,127]. However, the results in this study show that As concentrations in the leaves were toxic, which could indicate that detoxification mechanisms were not effective enough to prevent its accumulation in the leaves and potentially negative effects on plant function.

### 4.4. Chlorophyll a Fluorescence and Pigments Response to As Stress

Results in this study show lower values of photosynthetic efficiency (Fv/Fm), maximal (Fm), variable ChlF (Fv), and the ratio of Fm/Fo in *D. glomerata* sown on fly ash deposits versus the control site (78%, 72%, 58%, and 69%, respectively). In addition, values of Fv/Fm and Fm/Fo were below optimal range for plants indicating photoinhibition of PSII, a low PSII activity, and low vitality of plants sown on fly ash. Low values of Fv/Fm were measured in herbaceous plants, shrubs, and trees grown on fly ash deposits: *Cirsium arvense, Epilobium collinum, Crepis bienis, Eupatorium cannabinum, Verbascum phlomoides, Calamagrostis epigejos, Oenothera biennis, Festuca rubra* (0.429–0.691) [8,9,13,128], *Tamarix tetandra, Amorpha fruticosa* and *Spiraea van-houttei* (0.588–0.727), and *Populus alba* and *Robinia pseudoaccacia* (0.541–0.626) [8,129]. However, the values of minimal ChlF (Fo) did not differ between sites, whereas the values of t_1/2_ were higher compared to the control site (123%) indicating a lower sensitivity of these fluorescence kinetic parameters to stress suggesting induction of photoprotective mechanisms in order to maintain the PSII photochemistry as high as possible.

Results in the present study show that As in leaves significantly lowered the values of Fv/Fm, Fm and Fv (r = −0.71, r = −0.75, r = −0.73, respectively), indicating the adverse effects of As on PSII activity. Likewise, [13] reported that excess As in the leaves of *F. rubra* sown on fly ash deposit decreased Fv/Fm, Fm and Fv. Other authors also confirmed that As decreased Fv/Fm in plants, such as *Zea mays* [37], *Avena sativa* [39], *Hydrilla verticillata* [130] and *Nostoc muscorum* [131]. Reduced values of Fv/Fm under As exposure indicate a decrease in the maximum quantum yield of primary PSII chemistry and the low number of functionally active PSII centers. A low efficiency of photosynthesis (Fv/Fm) is related to decreased values of Fm (r = 0.99) and Fv (r = 0.99) suggesting the impairment of PSII activity at the donor side, significant accumulation of reduced primary quinone acceptor of PSII (Q_A_) and an increased number of closed PSII reaction centers that are unable to perform photochemistry. In addition, reduction in Fv parameter indicates a decrease in non-photochemical quenching of excess energy due to a lower amount of carotenoids (r = 0.90). However, results in this study show that the minimal ChlF (Fo) and t_1/2_ were enhanced under As stress (r = 0.52 and r = 0.90, respectively) suggesting that energy transfer from light harvesting complex (LHCII) to the PSII reaction center is efficient with no inactivation/damage in OEC (oxygen-evolving complex) enabling electron flow from primary quinone acceptors (Q_A_) to the secondary quinone acceptors (Q_B_), transferring electrons to the PSI which all together lead to the recovery of PSII photochemistry (r = 0.51 and r = 0.82, respectively).

Our results show that reduced content of Chl *a*, Chl *b*, Chl *a+b*, the ratio of Chl *a/b*, and total carotenoids in the leaves of *D. glomerata* sown on fly ash deposits in relation to the control site (66%, 53%, 63%, 82%, and 75%, respectively) can be the result of toxic As concentrations in leaves (r = −0.88, r = −0.85, r = −0.87, r = −0.95, and r = −0.84, respectively) indicating high sensitivity of photopigments to As stress. As decreased chlorophylls and carotenoids content in *Zea mays* [37], *Avena sativa* [39], *Oryza sativa* [132], *Sesuvium portulacastrum* [133], *Hydrilla verticillata* [130], *Pistia stratoides* [134], *Lemna gibba* [135], *Festuca rubra* [13], *Nostoc muscorum* [131], whereas [136] *Lactuca sativa* did not show any changes in the concentrations of photopigments under As stress. Generally, Chl *a* builds the light harvesting pigment complex (LHCP complex) and the core of photochemical reaction centers (PSI-P700 and PSII-P680) whereas Chl *b* is the accessory pigment which can absorb light and transfer energy to the other pigment molecules [137,138]. A decline in the Chl *a* and Chl *b* content in this study may be due to inhibition/reduction activity of enzymes that participates in chlorophyll synthesis, such as δ-aminolevulinic acid (ALA)-dehydrogenase and protochlorophylide reductase [139], or replacement of Mg atom with metals [140]. Furthermore, reduced carotenoids content in this study can disable an effective transfer of energy to Chl *a* and decreased protection of the photosynthetic aparatus from oxidative destruction, i.e., As decreases dissipation of excess energy in the form of heat.

Reduction in carotenoids content may also disable removing the triplet excited state of chlorophylls (^3^Chl*) and the singlet oxygen (^1^O_2_) [141]. Therefore, reduction in chlorophylls and carotenoids content results in a decrease of overall photosynthetic efficiency (Fv/Fm), which can be observed in our study as positive correlations between Fv/Fm, Fm, Fv and chlorophylls/carotenoids (Table S1). However, positive correlations between Fo, t_1/2_ and chlorophylls/carotenoids (Table S1) may suggest that the loss of the photoactive PSII reaction centers and antenna pigments can be ameliorated by some protection mechanisms that provide efficient electron transfer and recovery of PSII photochemistry.

In the leaves of *D. glomerata* sown on fly ash deposits, the amount of anthocyanins was lower than at the control site (71%) and it significantly decreased with high As content (r = −0.77). Unlike our results, As enhanced production of anthocyanins in *Azzola caroliliana* [142], *Lemna gibba* [134] and *Festuca rubra* grown on fly ash deposits [13]. According to [143], the redox status of the plastoquinone (PQ) pool regulates anthocyanin biosynthesis genes. Thus, positive correlation between t_1/2_ and anthocyanins in our study (r = 0.89) may suggest that high pool size of electron acceptors activates regulatory complex that is required for expression of key genes for anthocyanin accumulation, such as COP1 (CONSTITUTIVE PHOTOMORPHOGENIC1) and MYB transcription factors PRODUCTION OF ANTHOCYANIN PIGMENT1 (PAP1) and PAP2 [144,145]. Furthermore, anthocyanins can protect photosynthetic tissues from photooxidation by reducing the amount of available light and excitation pressure [146]. Thus, negative correlations between anthocyanins and Fv/Fm, Fm and Fv (r = −0.94, r = −0.94, and r = −0.93, respectively) may suggest that intense depletion of anthocyanins can decrease PSII inactivity to a certain extent.

**Figure 2:**
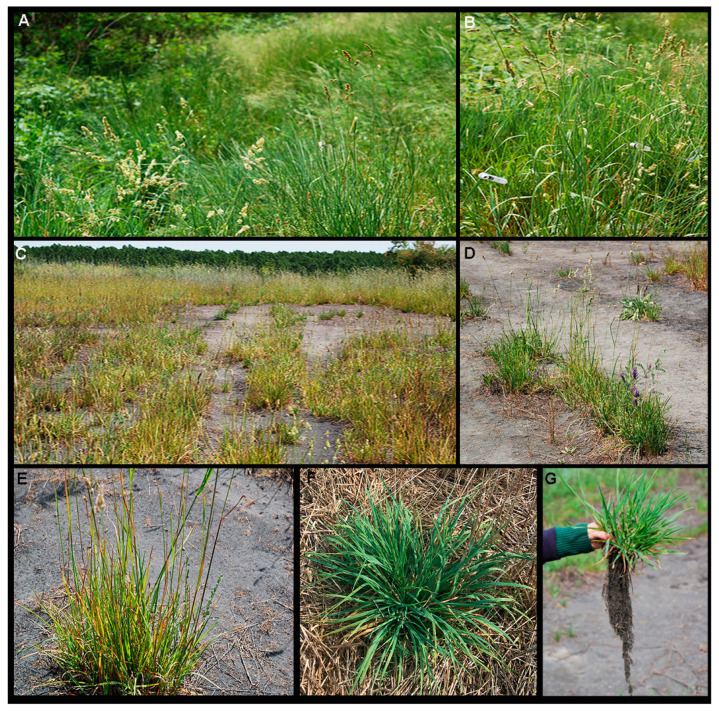
*D. glomerata* grown at the control site (CS) (**A**,**B**); *D. glomerata* grown at the fly ash site (L3) (**C**,**D**); Visible leaf damages of *D. glomerata* grown at the fly ash site (L3) (**E**); Green and vital leaves of *D. glomerata* grown at the fly ash site (L3) (**F**); Fibrous root system of *D. glomerata* grown at the fly ash site (L3) (**G**).

Visible leaf damages, such as chloroses and necroses, are similar to the deficient/excess of various chemical elements or those related to drought, and they are a direct consequence of changes in plant metabolic processes [40,45]. Thus, concentrations of chemical elements in the soil and in the plants, and determination of physiological and biochemical changes (photosynthesis, content of pigments) are necessary in the early assessment of visible symptoms of toxicity of chemical elements [45]. Therefore, leaves of *D. glomerata* grown at the control site were green color, without visual symptoms of damage (Figure 2A,B), which coincided with low total and available As, B, Cu, Mo, and Se concentrations in soils and plant tissues, as well as with high photosynthetic efficiency and content of pigments. Morphological damages of leaves in *D. glomerata* sown on fly ash deposits were in the form of chloroses light green and yellow color and necroses violet, red, and brown color (Figure 2C–E) that may be the result of toxic concentrations of As [40]. This plant species is exposed to low available B, Cu, Mo, and Se concentrations in fly ash and in the plant tissues. However, reduced photosynthetic efficiency and low concentrations of chlorophylls and carotenoids are related to excess As content in fly ash and plant leaves. Additionally, leaf chloroses and necroses of *D. glomerata* grown on fly ash may be the result of the synergistic effect of drought and toxic As concentrations. Furthermore, there is a large number of individuals of *D. glomerata* sown on fly ash deposits with green leaves (Figure 2F) and with a well-developed fibrous root system (Figure 2G), suggesting that despite excess As in leaves, this plant species is capable of activating protective mechanisms which raise its level of photosynthesis and vitality on fly ash deposits.

### 4.5. Oxidative Stress and Antioxidant Response to As Stress

Results in the present study show that an increase of MDA content in *D. glomerata* sown on fly ash deposit compared to the control site (115%) can be associated with high As concentrations in leaves (r = 0.85). High concentrations of As increased the amount of MDA in the leaves of *Festuca rubra* [13], *Zea mays* [37], *Oryza sativa* [38], *Adianthum capilillus-veneris* [147] and *Vigna mungo* [126]. Arsenic leads to lipid peroxidation of chloroplast membranes, increases their permeability and reduces the amount of pigments and affects photosynthesis [148] which is in our study confirmed by negative correlations between MDA and Fv, Fm, Chl *a*, Chl *b*, Chl *a+b*, Carot (r = −0.92, r = −0.92, r = −0.95, r = −0.95, r = −0.95, and r = −0.97, respectively). However, positive correlations between MDA and Fv/Fm, t_1/2_ and Fo (r = 0.90, r = 0.91 and r = 0.50, respectively) indicate that with high concentration of MDA, high PQ pool size and electron flow can lead to high photosynthetic efficiency. At higher pH of cytosol, chemical reactivity of MDA is small and production of a large amount of it can be adaptive response, i.e., MDA can act as the buffer of ROS with a protective function where the large amount of MDA can be a signal for the gene expression of antioxidant enzymes and molecules during the oxidative stress [35,36,149,150].

In the leaves of *D. glomerata* sown on fly ash deposits, the amount of free, bound and total phenolics was higher in comparison to the control site (137%, 140% and 137%, respectively) and it can be a response to As stress (r = 0.66, r = 0.76, and r = 0.66, respectively), indicating that As can promote their biosynthesis. Similar to our results, increased amount of phenolics under excess As in leaves was found in the leaves of *Festuca rubra* sown on fly ash deposits [13] and *Camellia sinesis* [151]. Results in this study show positive correlations between MDA and free, bound and total phenolics (r = 0.80, r = 0.93, and r = 0.82, respectively), suggesting that a high amount of MDA can be a signal for the activation of phenolics as antioxidants in order to maintain cellular redox homeostasis. Accumulation of phenolics in photosynthetic tissue of plants can reduce photoinhibition of PSII [152] which in our study is noted as positive correlations between free, bound and total phenolics and Fv/Fm, t_1/2_ and Fo (Table S1).

Furthermore, in the leaves of *D. glomerata* sown on fly ash deposits, the amount of AsA was higher (218%) compared to the control site and it is related to excess As (r = 0.72). Increased content of AsA has been observed in the condition of excess As in the leaves of *Festuca rubra* [13], *Cucumis sativus* [153], *Albizia procera* [154] and *Shorea robusta* [155]. Positive correlation between MDA and AsA (r = 0.90) suggests that high content of MDA can act as a signal for activation of ascorbic acid biosynthesis as a key player in redox hub. Results in this study show positive correlations between AsA and Fv/Fm, t_1/2_ and Fo (r = 0.99, r = 0.83, and r = 0.51, respectively), indicating that ascorbate can alleviate the inactivation of reactive centers of PSII by acting as an alternative donor to the P_680_^+^ and Tyr_Z_ [156]. In the current study, content of free phenolics increased with high content of AsA (r = 0.95). It has been reported that ascorbate is a cofactor for enzyme hydroxylases which are involved in the biosynthesis of flavonoids [157].

Radical scavenging activity in the leaves of *D. glomerata* sown on fly ash deposits was lower compared to the control site (61%) and that can be the result of excess As in leaves (r = −0.88). The total antioxidant capacity (DPPH) and MDA in the leaves was positively correlated (r = 0.95) suggesting that greater content of MDA can be a signal for increasing antioxidant biosynthesis which is crucial for detoxification of metal(loid)s. However, negative correlations between DPPH antioxidant activity and free, bound and total phenolics, and AsA (r = −0.85, r = −0.83, r = −0.86, and r = −0.90, respectively) can be attributed to the depletion of these antioxidants in increasing overall photosynthetic efficiency (AsA/Fv/Fm, AsA/t_1/2_ and AsA/Fo: r = −0.90, r = −0.88, r = −0.38, respectively).

In the roots of *D. glomerata*, MDA content was similar on both sites, whereas, concentrations of free, bound, and total phenolics, AsA and DPPH radical scavenging activity was greater compared to the control site (196%, 303%, 253%, 286% and 117%, respectively) which can be stress response to high As content (r = 0.96, r = 0.96, r = 0.97, r = 0.52, and r = 0.92, respectively). Furthermore, greater As content in roots decreased the content of MDA (r = −0.80), and that can be attributed to greater content of free, bound and total phenolics, AsA and total antioxidant DPPH activity (r = −0.87, r = −0.87, r = −0.87, r = −0.67, and r = −0.81, respectively), indicating that *D. glomerata* sown on fly ash deposits has high capacity to promote antioxidant biosynthesis as a response to As stress.

## 5. Conclusions

This study shows that fly ash deposits are characterized by alkaline pH reaction, lower content of organic matter, higher concentrations of available P_2_O_5_, higher total and available As, B, Cu, Mo, and Se concentrations (except Cu _available_) and higher As, B, Cu, Mo, and Se content in roots and leaves (except Cu _leaf_) compared to the control site. Generally, small available element concentrations in the fly ash and effective activation of B, Cu, Mo and Se mechanisms of acquisition, transportation and metabolisms have contributed to that *D. glomerata* limits their accumulation in leaves to the safe level i.e., values did not exceed the optimal range in leaves. However, *D. glomerata* sown on fly ash deposits is an excluder plant because it largely retains As in roots rather than in leaves (BCF < 1 and TF < 1) which points out that this grass possesses a high phytostabilization potential for As. Results in the present study could indicate that Pi application may reduce As uptake and transport into plant tissue. The proposed area size under As that *D. glomerata* can phytostabilized is one that has been biorecultivated by the original grass-legume mixture. Furthermore, toxic As concentrations in leaves reduced PSII photosynthetic efficiency, and chlorophyll/carotenoids/anthocyanins content indicating the sensitivity of PSII activity and pigment content to As stress. However, high MDA content may act as a signal for activation of antioxidant biosynthesis increasing the content of phenolics, ascorbic acid and total scavenging capacity, which in turn provides efficient electron transfer and recovery PSII photochemistry. Results also show that As in the roots increased biosynthesis of antioxidants reducing oxidative stress. Our findings indicate that *D. glomerata* possesses a high ecophysiological adaptive potential which enables it to grow and survive on As contaminated sites. Finally, *D. glomerata* can be considered as suitable plant for sustainable phytoremediation offering great prospects in phytomanagement of fly ash deposits and efficient policy frameworks to promote “green” cleanup of As-polluted sites worldwide.

## Figures and Tables

**Table 1:** Chemical properties, total and available element concentration in soil/fly ash, element concentration in plant root/leaf, bioconcentration factor (BCF) and translocation factor (TF) of *D. glomerata* growing at the control site (CS) and the fly ash site (L3).

Parameters	CS		L3		^a^ Range
	M (SD)	Min.–Max.	M (SD)	Min.–Max.	
pH (H_2_O)	7.59 (0.039)	7.54–7.68	7.92 (0.117) ***	7.78–8.12	
Org. matter (%)	5.83 (1.193) ***	4.11–7.02	3.13 (0.269)	2.75–3.59	
P_2_O_5_ (mg/100 g)	13.47 (4.853)	6.74–19.3	20.44 (2.256) ***	18.22–25.32	
As _Tot_ (μg/g)	7.28 (0.375)	6.26–8.08	20.13 (3.065) ***	16.41–23.98	4.4–9.3
B _Tot_ (μg/g)	4.51 (1.401)	2.91–6.99	49.54 (9.265) ***	33.97–60.74	22.0–45.0
Cu _Tot_ (μg/g)	15.78 (2.398)	13.25–18.50	49.99 (4.008) ***	40.41–53.98	13.0–24.0
Mo _Tot_ (μg/g)	1.12 (0.087)	0.92–1.23	1.82 (0.157) **	1.45–1.98	0.7–1.5
Se _Tot_ (μg/g)	0.25 (0.055)	0.14–0.33	2.32 (0.631) ***	1.82–3.75	0.25–0.34
As _avail._ (μg/g)	0.153 (0.029)	0.117–0.189	0.346 (0.139) ***	0.222–0.621	
B _avail._ (μg/g)	0.333 (0.110)	0.220–0.450	1.458 (0.269) ***	1.160–1.794	
Cu _avail._ (μg/g)	1.274 (0.413) **	0.867–1.700	0.893 (0.049)	0.818–0.975	
Mo _avail._ (μg/g)	0.008 (0.001)	0.007–0.011	0.024 (0.005) ***	0.018–0.032	
Se _avail._ (μg/g)	0.019 (0.004)	0.012–0.025	0.033 (0.012) **	0.014–0.050	
As _root_ (μg/g)	4.44 (0.539)	3.25–5.00	8.08 (0.881) ***	6.75–9.51	
B _root_ (μg/g)	5.13 (1.718)	3.25–6.88	16.15 (2.492) ***	12.89–19.65	
Cu _root_ (μg/g)	6.45 (0.271)	6.12–6.75	10.37 (1.383) ***	8.25–12.39	
Mo _root_ (μg/g)	0.77 (0.038)	0.74–0.85	0.95 (0.120) ***	0.75–1.12	
Se _root_ (μg/g)	2.45 (0.584)	1.25–3.50	5.53 (1.563) ***	2.87–7.38	
As _leaf_ (μg/g)	3.37 (0.243)	3.00–3.75	6.85 (0.209) ***	6.38–7.13	1.0–1.7 (O); 5.0–20.0 (T)
B _leaf_ (μg/g)	3.50 (0.281)	3.12–4.12	43.61 (5.399) ***	35.25–50.40	10–100 (O); 50–200 (T)
Cu _leaf_ (μg/g)	8.54 (0.909) ***	7.50–10.45	6.22 (0.912)	5.00–7.37	5–30 (O); 20–100 (T)
Mo _leaf_ (μg/g)	2.73 (0.336)	2.37–3.12	3.22 (0.455) **	2.75–3.75	0.2–5.0 (O); 10–50 (T)
Se _leaf_ (μg/g)	1.63 (0.689)	0.62–2.62	3.49 (0.315) ***	3.00–3.87	0.01–2.0 (O); 5–30 (T)
As (BCF)	0.86 (0.088) ***	0.74–1.03	0.41 (0.086)	0.27–0.51	
B (BCF)	1.13 (0.106) ***	0.98–1.27	0.34 (0.122)	0.21–0.57	
Cu (BCF)	0.41 (0.066)	0.33–0.49	0.68 (0.186) ***	0.46–0.88	
Mo (BCF)	0.68 (0.085) ***	0.60–0.88	0.52 (0.085)	0.40–0.65	
Se (BCF)	10.65 (5.334) ***	4.89–24.09	2.42 (0.696)	1.57–4.01	
As (TF)	0.77 (0.131) **	0.63–1.07	0.61 (0.074)	0.47–0.71	
B (TF)	0.74 (0.220)	0.49–1.07	2.71 (0.241) ***	2.37–3.01	
Cu (TF)	1.32 (0.133) ***	1.18–1.54	0.62 (0.166)	0.43–0.84	
Mo (TF)	3.57 (0.548)ns	2.77–4.17	3.47 (0.840)	2.44–4.83	
Se (TF)	0.74 (0.465)ns	0.17–1.69	0.67 (0.198)	0.46–1.04	

ANOVA, Data represent means (SD); Min.—minimum values; Max.—Maximum values; n = 10, ** *p* < 0.01; *** *p* < 0.001; ns = not significant; ^a^ [40] O—optimal range of element concentrations; T—toxic range of element concentrations.

**Table 2:** Chlorophyll *a* fluorescence parameters, content of malondialdehyde (MDA, nmol/g), content of chlorophylls (mg/g), total carotenoids (mg/g), anthocyanins (mg/g), phenolics (mg/g), ascorbic acids (AsA, mg/g), and radical scavenging activity (DPPH, %) in leaves and roots of *D. glomerata* growing at the control site (CS) and the fly ash site (L3).

Parameters	CS		L3	
	M (SD)	Min.–Max.	M (SD)	Min.–Max.
**Chl *a* fluorescence**				
Fo	0.21 (0.259) ns	0.17–0.25	0.22 (0.014)	0.21–0.25
Fm	1.07 (0.305) *	0.30–1.32	0.77 (0.370)	0.39–1.20
Fv	0.95 (0.128) **	0.74–1.08	0.55 (0.371	0.19–0.97
t_1/2_	118.00 (17.770)	83.00–139.00	144.70 (12.11) ***	125.00–166.00
Fv/Fm	0.814 (0.009) ***	0.804–0.833	0.633 (0.179)	0.450–0.815
Fm/Fo	5.04 (1.378) *	1.20–6.10	3.50 (1.733)	1.80–5.36
**Pigments**				
Chl *a*	6.49 (1.291) ***	4.36–8.59	4.30 (0.940)	2.54–5.62
Chl *b*	2.09 (0.308) ***	2.09–0.30	1.12 (0.229)	0.66–1.39
Chl *a+b*	8.58 (1.346) ***	6.73–10.85	5.42 (1.147)	3.21–6.92
Chl *a/b*	3.81 (0.175) **	1.61–3.84	3.14 (0.619)	3.52–4.14
Tot Carot	1.77 (0.217) ***	1.27–1.99	1.33 (0.242)	0.88–1.61
Anthocyanins	1.270 (0.177) **	0.990–1.620	0.905 (0.269)	0.555–1.250
**Oxidative stress (L)**				
MDA	0.71 (0.108)	0.44–1.14	0.82 (0.128) *	0.66–1.00
**Antioxidants (L)**				
Free Phenolics	13.56 (5.779)	6.0–25.2	18.16 (5.468) *	7.30–36.60
Bound Phenolics	11.45 (2.623)	7.1–15.0	16.04 (3.380) **	12.90–22.00
Tot Phenolics	25.01 (10.073)	13.2–39.4	34.20 (14.560) *	20.40–57.30
AsA	0.49 (0.051)	0.43–0.57	1.07 (0.606) **	0.41–1.70
DPPH	24.22 (6.807) ***	14.32–30.46	14.75 (3.114)	10.85–19.58
**Oxidative stress (R)**				
MDA	0.42 (0.033) ns	0.37–0.47	0.44 (0.195)	0.22–0.73
**Antioxidants (R)**				
Free Phenolics	0.81 (0.078)	0.72–0.96	1.59 (0.353) ***	1.06–2.04
Bound Phenolics	0.91 (0.065)	0.82–0.99	2.76 (0.811) ***	1.52–3.75
Tot Phenolics	1.72 (0.140)	1.54–1.95	4.35 (1.163) ***	2.58–5.80
AsA	0.22 (0.068)	0.14–0.31	0.63 (0.192) ***	0.40–0.98
DPPH	37.58 (4.011)	30.45–43.67	44.15 (7.244) **	32.76–52.98

ANOVA, Data represent means (SD); Min.—minimum values; Max.—Maximum values; n = 10; * *p* < 0.05, ** *p* < 0.01; *** *p* < 0.001, ns = not significant; L—leaves; R—roots.

**Table 3:** The Pearson correlation coefficient (r) between parameters of chemical properties of fly ash and soil, total and available As concentrations in fly ash and soil, and As concentrations in roots and leaves of *D. glomerata* growing at fly ash deposits (FA) and soil at the control site (CS).

**FA/FA**	**r**	**FA/Plant**	**r**	**Plant/Plant**	**r**
pH/As _tot_	0.91	pH/As _root_	0.96	As _root_/As _leaf_	0.92
pH/As _avail_	0.95	pH/As _leaf_	0.88		
Org.mat./As _tot_	0.95	Org.mat./As _root_	0.98		
Org.mat./As _avail._	0.96	Org.mat./As _leaf_	0.90		
P_2_O_5_ /As _tot_	−0.84	P_2_O_5_/As _root_	−0.92		
P_2_O_5_ /As _avail._	−0.76	P_2_O_5_/As _leaf_	−0.93		
As _tot_/As _avail._	0.95	P_2_O_5_/BCF	−0.74		
		P_2_O_5_/TF	−0.85		
		As _tot_/As _root_	0.93		
		As _tot_/As _leaf_	0.80		
		As _avail._/As _root_	0.94		
		As _avail._/As _leaf_	0.78		
**CS/CS**	**r**	**CS/Plant**	**r**	**Plant/Plant**	**r**
pH/As _tot_	−0.06	pH/As _root_	−0.05	As root/As leaf	−0.13
pH/As _avail._	−0.69	pH/As _leaf_	0.24		
Org.mat./As _tot_	0.19	Org.mat./As _root_	0.33		
Org.mat./As _avail._	−0.75	Org.mat./As _leaf_	−0.08		
P_2_O_5_/As _tot_	0.03	P_2_O_5_/As _root_	0.21		
P_2_O_5_/As _avail._	−0.22	P_2_O_5_/As _leaf_	0.01		
As tot/As _avail._	0.26	P_2_O_5_/BCF	0.21		
		P2O5/TF	−0.20		
		As _tot_/As _root_	0.28		
		As _tot_/As _leaf_	0.22		
		As _avail._/As _root_	−0.14		
		As _avail._/As _leaf_	0.23		

**Table 4:** The Pearson correlation coefficient (r) between As concentrations in leaves and Chlorophyll *a* fluorescence (ChlF) parameters, content of pigments, MDA and antioxidants as well as the Pearson correlation coefficient (r) between As concentrations in roots and content of MDA, and antioxidants in *D. glomerata* growing at fly ash deposits (FA) and soil at the control site (CS).

**Leaves**	**FA**	**CS**	**Roots**	**FA**	**CS**
	**r**	**r**		**r**	**r**
As/Fv/Fm	−0.71	−0.17	As/MDA	−0.80	−0.01
As/Fm	−0.75	−0.08	As/Free Ph	0.96	0.08
As/Fv	−0.73	−0.16	As/Bound Ph	0.96	0.02
As/Fo	0.52	−0.15	As/Tot Ph	0.97	0.06
As/t1/2	0.90	−0.10	As/AsA	0.52	−0.35
As/Fm/Fo	0.71	−0.02	As/DPPH	0.92	−0.13
As/Chl *a*	0.88	−0.31			
As/Chl *b*	0.85	0.45			
As/Chl *a+b*	0.87	−0.18			
As/Chl *a/b*	0.95	−0.53			
As/Tot Carot	−0.84	−0.14			
As/Anthocy	−0.77	−0.21			
As/MDA	0.95	−0.44			
As/Free Ph	0.66	−0.10			
As/Bound Ph	0.76	−0.06			
As/Tot Ph	0.66	−0.14			
As/AsA	0.72	0.45			
As/DPPH	−0.86	0.01			

## Supplementary Materials

The following are available online at https://www.mdpi.com/2223-7747/9/5/657/s1, Table S1: The Pearson correlation coefficient (r) between Chlorophyll *a* fluorescence (ChlF) parameters, pigments (P), content of MDA and antioxidants (A) in the leaves of *D. glomerata* sown on fly ash deposits (L3). Table S2: The Pearson correlation coefficient (r) between content of pigments (P), MDA and antioxidants (A) in the leaves of *D. glomerata* sown on fly ash deposits (L3). Table S3: The Pearson correlation coefficient (r) between content of MDA and antioxidants (A) in the roots of *D. glomerata* sown on fly ash deposits (L3).
