# Supplementary material for: Phytoremediation Potential, Photosynthetic and Antioxidant Response to Arsenic-Induced Stress of Dactylis glomerata L. Sown on Fly Ash Deposits
Source: Plants (Basel). 2020 May 22;9(5):657. doi: 10.3390/plants9050657 (PMC7284476; doi:10.3390/plants9050657)
Supplement: Supplementary file 1 [file plants-09-00657-s001.pdf]

Supplementary Materials:

Table S1. The Pearson correlation coefficient (r) between Chlorophyll *a* fluorescence (ChlF) parameters, pigments (P), content of MDA and antioxidants (A) in the leaves of *D. glomerata* sown on the fly ash deposits (L3).

**Leaf**					
**ChlF / ChlF**	**r**	**ChlF / P**	**r**	**ChlF / MDA, A**	**r**
Fv/Fm / $t_{1/2}$	0.82	Fv/Fm / Chl *a*	0.82	Fv/Fm / MDA	0.90
Fv/Fm / Fm	0.99	Fv/ Fm / Chl *b*	0.86	Fv/Fm / Free Ph	0.95
Fv/Fm / Fo	0.51	Fv/Fm / Chl *a+b*	0.84	Fv/Fm / Bound Ph	0.85
Fv/Fm / Fv	0.99	Fv/Fm / Chl *a/b*	0.74	Fv/Fm / Tot Ph	0.94
Fv/Fm / Fm/Fo	0.99	Fv/Fm / Carot	0.90	Fv/Fm / AsA	0.99
$t_{1/2}$ / Fm	−0.84	Fv/Fm / Anthocy	−0.94	Fv/Fm / DPPH	−0.90
$t_{1/2}$ / Fo	0.49	$t_{1/2}$ / Chl *a*	0.97	$t_{1/2}$ / MDA	0.91
$t_{1/2}$ / Fv	−0.82	$t_{1/2}$/ Chl *b*	0.95	$t_{1/2}$ / Free Ph	0.80
$t_{1/2}$ / Fm/Fo	0.83	$t_{1/2}$ / Chl *a+b*	0.97	$t_{1/2}$ / Bound Ph	0.91
Fm / Fo	−0.47	$t_{1/2}$ / Chl *a/b*	0.95	$t_{1/2}$ / Tot Ph	0.83
Fm / Fv	0.99	$t_{1/2}$ / Carot	0.94	$t_{1/2}$ / AsA	0.83
Fm / Fm/Fo	−0.99	$t_{1/2}$ / Anthoc	0.89	$t_{1/2}$ / DPPH	−0.88
Fv / Fo	−0.47	Fm / Chl *a*	0.83	Fm / MDA	−0.92
Fv / Fm /Fo	−0.99	Fm / Chl *b*	0.97	Fm / Free Ph	−0.95
Fo / Fm/Fo	0.53	Fm / Chl *a+b*	0.85	Fm / Bound Ph	−0.86
		Fm / Chl *a/b*	−0.77	Fm / Tot Ph	−0.95
		Fm / Carot	0.91	Fm / AsA	−0.99
		Fm / Anthoc	−0.94	Fm / DPPH	0.93
		Fv / Chl *a*	0.82	Fv / MDA	−0.92
		Fv / Chl *b*	0.86	Fv / Free Ph	−0.95
		Fv / Chl *a+b*	0.84	Fv / Bound Ph	−0.84
		Fv / Chl *a/b*	−0.75	Fv / Tot Ph	−0.94
		Fv / Carot	0.90	Fv / AsA	−0.99
		Fv / Anthoc	−0.93	Fv / DPPH	0.93
		Fo / Chl *a*	−0.58	Fo / MDA	0.50
		Fo / Chl *b*	−0.67	Fo / Free Ph	0.41
		Fo / Chl *a+b*	−0.60	Fo / Bound Ph	0.61
		Fo / Chl *a/b*	0.57	Fo / Tot Ph	0.40
		Fo / Carot	−0.56	Fo / AsA	0.51
		Fo / Anthoc	−0.51	Fo / DPPH	−0.38
		Fm /Fo / Chl *a*	−0.83	Fm/Fo / MDA	0.91
		Fm /Fo / Chl *b*	−0.87	Fm/Fo / Free Ph	0.95
		Fm /Fo / Chl *a+b*	−0.85	Fm/Fo / Bound Ph	0.87
		Fm /Fo / Chl *a/b*	0.75	Fm/Fo / Tot Ph	0.95
		Fm /Fo / Carot	−0.90	Fm/Fo / AsA	0.99
		Fm/Fo / Anthoc	−0.95	Fm/Fo / DPPH	−0.89

Table S2. The Pearson correlation coefficient (r) between content of pigments (P), MDA and antioxidants (A) in leaves of *D. glomerata* sown on the the fly ash deposits (L3).

Leaf					
**P / MDA, A**	**r**	**MDA / A**	**r**	**A / A**	**r**
Chl *a* / MDA	−0.95	MDA / Free Ph	0.80	Free Ph / Bound Ph	0.80
Chl *a* / Free Ph	−0.74	MDA / Bound Ph	0.93	Free Ph / Tot Ph	0.99
Chl *a* / Bound Ph	−0.95	MDA / Tot Ph	0.82	Free Ph / AsA	0.95
Chl *a* / Tot Ph	−0.77	MDA / AsA	0.90	Free Ph / DPPH	−0.85
Chl *a* / AsA	−0.82	MDA / DPPH	0.95	Bound Ph / Tot Ph	0.83
Chl *a* / DPPH	0.87			Bound Ph / AsA	0.85
Chl *b* / MDA	−0.95			Bound Ph / DPPH	−0.83
Chl *b* / Free Ph	−0.79			Tot Ph / AsA	0.95
Chl *b* / Bound Ph	−0.96			Tot Ph / DPPH	−0.86
Chl *b* / Tot Ph	−0.82			AsA / DPPH	−0.90
Chl b / AsA	−0.86				
Chl *b* / DPPH	0.88				
Chl *a+b* / MDA	−0.95				
Chl *a+b* / Free Ph	−0.77				
Chl *a+b* / Bound Ph	−0.96				
Chl *a+b* / Tot Ph	−0.81				
Chl *a+b* / AsA	−0.84				
Chl *a+b* / DPPH	0.88				
Chl *a/b* / MDA	0.89				
Chl *a/b* / Free Ph	0.70				
Chl *a/b* / Bound Ph	0.88				
Chl *a/b* / Tot Ph	0.72				
Chl *a/b* / AsA	0.75				
Chl *a/b* / DPPH	−0.87				
Carot / MDA	−0.97				
Carot / Free Ph	−0.84				
Carot / Bound Ph	−0.96				
Carot / Tot Ph	−0.87				
Carot / AsA	−0.90				
Carot / DPPH	0.92				
Anthocy / MDA	−0.95				
Anthocy / Free Ph	−0.87				
Anthocy / Bound Ph	−0.95				
Anthocy / Tot Ph	−0.89				
Anthocy / AsA	−0.93				
Anthocy / DPPH	0.93				

Table S3. The Pearson correlation coefficient (r) between content of MDA and antioxidants (A) in roots of *D. glomerata* sown on the fly ash deposits (L3).

Root			
**MDA / A**	**r**	**A / A**	**r**
MDA / Free Ph	−0.87	Free Ph / Bound Ph	0.99
MDA / Bound Ph	−0.87	Free Ph / Tot Ph	0.99
MDA / Tot Ph	−0.87	Free Ph / AsA	0.62
MDA / AsA	−0.69	Free Ph / DPPH	0.98
MDA / DPPH	−0.81	Bound Ph / Tot Ph	0.99
		Bound Ph / AsA	0.55
		Bound Ph / DPPH	0.95
		Tot Ph / AsA	0.57
		Tot Ph / DPPH	0.96
		AsA / DPPH	0.57
